# How post overdose response efforts can address social determinants of health among people who use drugs: perspectives from the New York State Department of Health (NYSDOH) Post Overdose Response Team (PORT)

**DOI:** 10.1186/s12954-024-01081-z

**Published:** 2024-10-03

**Authors:** Danielle Lloyd, Nerissa George, Donald Grove, Rebecca Goldberg, Allan Clear

**Affiliations:** 1 Office of Drug User Health, AIDS Institute, New York State Department of Health, 90 Church Street, 13th Floor, New York, NY 10007 USA; 2 Office of Program Evaluation and Research, AIDS Institute, New York State Department of Health, Corning Tower, Room 342, Albany, NY 12237 USA; 3 Health Policy, Management and Behavior Department, University at Albany School of Public Health, 1 University Plaza, Rensselaer, NY 12144 USA; 4 Present Address: Vermont, USA

**Keywords:** Harm reduction, People who use drugs, Post overdose response team, Social determinants of health

## Abstract

Individuals who have survived an overdose often have myriad needs that extend far beyond their drug use. The social determinants of health (SDOH) framework has been underutilized throughout the opioid overdose crisis, despite widespread acknowledgment that SDOH are contributors to the majority of health outcomes. Post Overdose Response Teams (PORTs) engage with individuals who have experienced 1 or more nonfatal overdoses and bear witness to the many ways in which overdose survivors experience instability with healthcare, housing, employment, and family structure. Employing a harm reduction model, PORTs are well-positioned to reach people who use drugs (PWUD) and to address gaps in basic needs on an individualized basis, including providing social support and a sense of personal connection during a period of heightened vulnerability. The New York State Department of Health (NYSDOH) PORT program is a harm reduction initiative that utilizes law enforcement data and several public databases to obtain accurate referral information and has been active since 2019 in NYC. This PORT program offers various services from overdose prevention education and resources, referrals to health and treatment services, and support services to overdose survivors and individuals within their social network. This perspective paper provides an in-depth overview of the program and shares quantitative and qualitative findings from the pilot phase and Year 1 of the program collected via client referral data, interviews, and case note reviews. It also examines the barriers and successes the program encountered during the pilot phase and Year 1. The team’s approach to addressing complex needs is centered around human connection and working toward addressing SDOH one individualized solution at a time. Application of the NYSDOH PORT model as outlined has the potential to create significant positive impacts on the lives of PWUD, while potentially becoming a new avenue to reduce SDOH-related issues among PWUD.

## Background

### The overdose crisis and social determinants of health

In the United States, the broadening demographics of people who use drugs (PWUD) and the significant rise of opioid-related fatalities over the past decade, in large part due to the increase in illegally made fentanyl in the drug supply [6], have resulted in a public health crisis. In New York City (NYC) specifically, fentanyl was the most common opioid involved in overdose deaths, associated with 68% of overdose deaths in 2019, an increase of 8% from 2018 [14].

Increasingly, public health professionals argue that a more comprehensive approach to address root causes is necessary, calling for the social determinants of health framework to be adopted and applied to overdose prevention efforts [17, 8]. Although there is widespread acknowledgment that social and economic determinants of health continue to contribute to health disparities and inequities [21], many response efforts remain “too narrow for the scale and scope of the crisis.” [17].

Social determinants of health (SDOH) are defined as the “conditions in the environments where people are born, live, learn, work, play, worship, and age that affect a wide range of health, functioning, and quality-of-life outcomes and risks” [21]. Five domains broadly represent the SDOH, as described by the US Department of Health and Human Services Healthy People 2030 initiative: (1) economic stability; (2) education access and quality; (3) healthcare access and quality; (4) neighborhood and built environment; (5) social and community context. It is estimated that these determinants, which include socioeconomic and environmental factors as well as health-related behaviors, may be responsible for 80–90% of health outcomes and are of particular importance concerning behavioral health outcomes [4].

Van Draanen et al. [22] describe the effects of socioeconomic marginalization (SEM), the reality for many PWUD, and highlight the repercussions which include exclusion from the formal labor market, material insecurity, incarceration, social stigma, isolation, and poverty. Conditions that may contribute to SEM such as homelessness, stigma within healthcare systems, a recent release from prison, and Medicaid eligibility have been linked to overdose [10, 22]. Social factors including experiencing early childhood trauma may be amplified by structural racism and income inequality and are critical in shaping risks associated with drug initiation and future use [17]**.** The criminalization of people who use drugs leads them to encounter law enforcement at high frequencies. This results in the adoption of behaviors that will avoid contant law enforcement such as using alone, in secret, without proper supplies, and not calling 911 during an overdose. Unfortunately, the very measures PWUDs take to protect themselves from violent encounters with police put them at greater risk of overdose [7]. The synergistic impact of these disadvantages, resulting in sustained psychological and physiological stress levels, has far-reaching effects and requires a comprehensive, person-centered response. Designing and implementing community-based solutions outside the healthcare and addiction treatment system, as well as within it, are necessary to respond effectively to the opioid crisis [4]. As stated by Braveman et al. [4], the questions are no longer about whether social and economic factors are important influences on health, but rather what interventions are most effective in “activating health-promoting pathways and interrupting health-damaging ones.”

Public health professionals have long employed the social determinants of health framework although it has been underutilized throughout the opioid overdose epidemic, a response that has frequently focused on supply-side approaches such as controlling prescription opioids and arresting PWUD [17]. An increasing amount of evidence supports the claim that addressing the social determinants of health and working toward health equity will likely improve health outcomes associated with opioid use [5, 8, 19]. However, with the exception of harm reduction, there remains a dearth of holistic, person-centered programs that specifically address drug overdose health inequities [19].

### The role of Post Overdose Response Teams (PORTs)

Post overdose response teams (PORTs) have been identified as an emerging strategy to meaningfully engage with people who have experienced a non-fatal overdose. Such work has been carried out to reach PWUD in various capacities for decades, spearheaded by leaders in the grassroots harm reduction movement. Some of the earliest documented harm reduction efforts worked to stem the spread of HIV and hepatitis in the United Kingdom and Western Europe and utilized the skills of outreach workers who provided PWUD with support and connection to care [9, 15].

PORT services may vary, but overarching goals remain the same: to provide linkages to life-saving services such as medication for opioid use disorder (MOUD) and offer overdose education and counseling coupled with naloxone during a particularly vulnerable period following a non-fatal overdose incident. Most often, PORTs form as a result of cross-sector efforts between public health professionals or harm reduction organizations and first responders, such as emergency medical services or law enforcement. These teams may operate out of emergency departments, visit non-fatal overdose survivors at their homes or locations of overdose, or offer some combination of options [2].

Individuals who have survived an overdose often have complex needs that extend far beyond their drug use. PORT programs, employing a harm reduction model, are well-positioned to reach PWUD to ensure basic needs are met and offer social support to individuals during a period of heightened vulnerability.

## Methods

### New York State Department of Health (NYSDOH) Post Overdose Response Team (PORT)

The multi-pronged approach of the NYSDOH PORT developed as a result of the ongoing efforts of NYC RxStat, a public health/public safety collaboration aiming to reduce overdose deaths [1]. This collaborative, facilitated by the New York–New Jersey High-Intensity Drug Trafficking Area, was designed to address the needs of two distinct target groups: individuals who had recently overdosed and the next of kin of overdose decedents. RxStat brings together high-level representatives from New York City and the State’s public health and safety agencies to coordinate a shared responsibility for responding to the overdose epidemic by pooling data that can help to identify those at an increased risk of a fatal overdose. Since the 2012 inception of NYC RxStat, jurisdictions across the US have implemented similar partnerships based on the model [1].

PORTs often obtain data via law enforcement records that are automatically collected when police officers respond to nonfatal overdose calls. The information collected by the responding officer is then shared with a harm reduction-oriented public health agency in a timely manner. This data allows for an individual-level harm reduction intervention to occur during a period of heightened vulnerability for an already at-risk community. This model harnesses the power of cross-sector collaboration and data sharing to address access to evidence-based harm reduction interventions and other needed services. Similar to RxStat, this approach uses existing public safety data sources combined with a harm reduction approach to reach members of the target population [11].

The NYSDOH PORT, consisting of 3 outreach staff, 1 program manager, 1 clinician, and evaluation support from 1 data analyst and 1 evaluation specialist, provides services throughout the 5 boroughs of New York City, which includes Manhattan, Brooklyn, Queens, the Bronx, and Staten Island. The team receives referral information from the City of New York Police Department (NYPD) sourced via law enforcement reports collected by NYPD officers and the New York City Office of Chief Medical Examiner including name, phone number, physical address, and overdose location. Client information is shared unidirectionally by NYPD to the PORT. The PORT then follows Health Insurance Portability and Accountability (HIPAA) and the NYSDOH AIDS Institute’s confidentiality standards of privacy to ensure the highest protection of client information. The team also accepts secondary referrals, ensuring members of an individual’s social network can be connected to services if needed. When an outreach team member contacts the original referral provided, they may recommend others in their social network who might be interested in or benefit from PORT services. The outreach team member requests that the original referral inform the individual in their social network of this opportunity for connection, so they are made aware before receiving a phone call from the team.

Since the likelihood of overdose fatality increases after an initial overdose occurrence, the NYSDOH PORT attempts to contact each referral as soon as possible to assess the individual’s needs and connect them to supportive services.

#### Reaching those most at risk

NYPD shares weekly referral information via Excel with PORT’s data coordinator for processing and quality assurance before the entry into PORT’s database. Upon receiving a referral, the NYSDOH PORT attempts to contact the individual via phone calls and text messages. If initial phone contact attempts are unsuccessful, a team member will attempt a home visit. Historically, a home visit would be attempted after two failed phone calls. If the contact information is determined to be incorrect, the team will search public records and utilize a state database to identify recent contact information. This database stores data on Medicaid enrollees and the behavioral health population with any history of substance use services, psychiatric or substance use diagnosis, or psychotropic medication. Although the database is primarily used to locate up-to-date contact information, the team can access additional information on an individual’s substance use and mental health treatment through the database with a signed release.

Once contact is established and the individual expresses willingness to engage in services, a member of the team schedules a meeting date, time, and location to learn more about the individual’s goals. After the initial contact, the same team member remains connected with the individual based upon their stated needs and provides follow-up as necessary.

#### Offering what people need

This program was designed to follow the core tenets of harm reduction by supporting individual agency and validating subjective experiences while meeting people where they are. The team works toward this goal in both literal and figurative senses: meeting clients at or near their homes or places where they are most comfortable and providing support at any stage in their substance use journey.

Each outreach team member has lived substance use experience and has been trained in motivational interviewing techniques, increasing the likelihood of initiating and maintaining connections with prospective clients. The scope of services provided is dictated by the clients' needs, which are identified during an initial assessment and vary significantly based on each client's circumstances. All clients are offered post-overdose safety planning and counseling, naloxone kits, and linkages to a same-day buprenorphine provider. Depending on the client's stated needs, the team member will offer a connection to a provider in their community or facilitate buprenorphine induction with the team’s in-house clinician. Clients can access other types of MOUD, such as methadone, if preferred. Many clients decline MOUD but request support in other ways, which speaks to the value of the care coordination efforts the team undertakes. This includes warm handoffs to other providers such as social services, Drug User Health Hubs, primary care, mental health care (including bereavement services for those who recently lost a loved one), escorts to appointments and assistance with transportation.

Descriptive statistics were calculated to characterize the successes and challenges the NYSDOH PORT team experienced when performing outreach to clients referred by the NYPD. We examined the program’s ability to reach and serve clients. Qualitative analyses were performed on outreach members' case notes and bi-weekly qualitative interviews that were facilitated by the program’s evaluator.

## Results

### Overview of NYSDOH PORT referral data

This paper will be sharing an overview of NYPD referral data (Tables 1, 2). During the 12-month pilot phase (September 2019–August 2020) of the project, a total of 148 NYPD referrals were made to the PORT team. The team successfully contacted 53 individuals (36% of referrals), and 29 of those contacted accepted services (55% reached). After multiple contact attempts, 64% (95 out of 148) of referrals could not be reached. Approximately 22% (32 out of 148) of total referrals were female, and 34% (11 out of 32) of females in the referral pool were reached.

**Table 1:** PORT referral demographics: successful referral contacts

Variable	Pilot Year—*n*(%) (*n* = 53)	Year 1—*n*(%) (*n* = 204)
Age		
Range	23–76	14–78
Median	44	42
Sex		
Female	11 (20.8)	73 (35.8)
Male	42 (79.2)	131 (64.2)
Borough		
Bronx	17 (32.1)	57 (27.9)
Brooklyn	10 (18.9)	33 (16.2)
Manhattan	8 (15.1)	41 (20.1)
Queens	8 (15.1)	29 (14.2)
Staten Island	8 (15.1)	41 (20.1)
Unknown	2 (3.8)	3 (1.5)
Race		
Black	10 (18.9)	48 (23.5)
Hispanic	15 (28.3)	63 (30.9)
Asian/Pacific Islander	2 (3.8)	2 (1.0)
White	24 (45.3)	84 (41.2)
Unknown	2 (3.8)	7 (3.4)

**Table 2:** PORT referral demographics: referrals who accepted services

Variable	Pilot Year—*n*(%) (*n* = 29)	Year 1—*n*(%) (*n* = 124)
Age		
Range	23–76	14–78
Median	46	42.5
Sex		
Female	7 (24.1)	48 (38.7)
Male	22 (75.9)	76 (61.3)
Borough		
Bronx	14 (48.3)	39 (31.5)
Brooklyn	5 (17.2)	16 (12.9)
Manhattan	6 (20.7)	25 (20.2)
Queens	3 (10.3)	19 (15.3)
Staten Island	1 (3.4)	22 (17.7)
Unknown	0 (0)	3 (2.4)
Race		
Black	5 (17.2)	31 (25.0)
Hispanic	12 (41.4)	41 (33.1)
Asian/Pacific Islander	1 (3.4)	0 (0)
White	11 (37.9)	47 (37.9)
Unknown	0 (0)	5 (4.0)

During the first year of operations following the pilot phase (referred to as Year 1; September 2020–August 2021), there was a 645% increase in referrals compared to the pilot year. In Year 1, the team reached 21% (204 out of 954) of referrals. Nearly 61% (124 out of 204) of those reached accepted services. After multiple contact attempts, about 79% (750 out of 954) of individuals could not be reached. Roughly 25% (241 out of 954) of referrals were female, and 30% (72 out of 241) of females in the referral pool were reached.

Over the two time periods observed, there was a 4% decrease in female referrals reached, however, the volume of female referrals received increased by roughly 750%. The median number of outreach attempts to contact each referral decreased from 3 to 2 attempts. The median length of time it took for individuals to receive their respective service(s) remained at one day for both observed periods. During the pilot phase, most unreachable referrals were in the Bronx (23%), followed by Brooklyn (19%) and Queens (19%). During Year 1, most unreachable referrals were in Brooklyn (28%), followed by Queens (25%), and Manhattan (18%). During the pilot phase, referrals who received a home visit were less likely to accept services than those who did not receive a home visit (i.e., phone and text outreach only), 30% and 70%, respectively. However, after the pilot phase, referrals who received a home visit were more likely to accept services than those who did not receive a home visit, 79% and 21%, respectively.

### Findings from interviews with outreach workers

Based upon bi-weekly interviews with the NYSDOH PORT, several client-level and system-level barriers were identified that impeded the team’s ability to reach referrals and/or connect referrals to services. Some critical obstacles the team navigates are associated with client confidentiality. The team often has contact with individuals within the referral's social network, and many of these individuals are not aware of the referral's drug use or recent overdose occurrence. Since team members cannot inform the referral’s social network of the purpose of the home visit, the team member may only pass along a vague message with contact information, requesting the referral reach out to a member of the team. Additionally, inaccurate or incomplete referral phone numbers and addresses have proven to be significant barriers to reaching individuals.

Some of the most common system-level barriers the team has experienced when linking an individual to care are associated with provider accessibility and protocols. It can be a challenge to find a MOUD provider who is available the same day or in the nearer term and has open appointments on evenings and/or weekends in a convenient location. Many MOUD provider practices require a burdensome intake process to be completed, in addition to meeting on-site counseling requirements. Additional barriers include a lack of coordination with pharmacies to prescribe MOUD formulations covered by insurance and perceived risks associated with MOUD, which interferes with providing timely, appropriate overdose prevention and treatment.

Another significant challenge the team faces is specific to the number of referrals from this historically hard-to-reach population who are lost to follow-up. Team member morale has been adversely impacted by losing contact with individuals known to be at heightened risk of harm. Regular meetings with a clinical social worker are held to provide support to the team and prevent burnout.

Despite barriers, the team has reported numerous successes, including the distribution of naloxone kits and fentanyl test strips, providing overdose prevention education, and linking individuals to buprenorphine and outpatient treatment. During the pilot phase of the program, 8 naloxone kits were distributed, 20 referrals received overdose prevention education, 2 referrals were linked to buprenorphine, and 3 referrals were linked to outpatient treatment. In Year 1, 94 naloxone kits were distributed, 104 referrals received overdose prevention education, 5 referrals were linked to buprenorphine, and 16 referrals were linked to outpatient treatment. Fentanyl test strip distribution figures are not available during these periods because distribution did not begin until July 2022.

Additionally, the team has assisted in reducing the burden of multiple referrals, instead focusing on establishing warm hand-offs. For example, a team member referred a new client in need of MOUD to the team clinician. Previously, this client had received his prescription through a private provider but had high out-of-pocket expenses. After connecting the client with the team clinician as a temporary solution, he was further assisted with a linkage to a long-term MOUD provider with lower out-of-pocket costs. There have also been instances in which new clients were prescribed MOUD on the same day a home visit was performed, highlighting the importance of having a prescribing provider on the team.

### Findings from case note review

Chris, Samantha, and Ezra, whose stories are below, were all referred to NYSDOH PORT following their own or a loved one’s overdose. They were all at elevated risk of overdose, based on the information shared with the team during their initial assessment. Their stories are representative of the complex needs of many PWUDs. Their stories also highlight the interplay of overdose risk and housing, employment, relationships, healthcare, and environmental factors.

#### Chris

An example of reducing the risk of mortality by addressing a pain point in a person’s SDOH can be understood through one of PORT’s clients, “Chris”. Chris was at risk of overdose for many reasons, but his primary stressors when the team engaged him included acute grief from the recent passing of his wife due to a fatal overdose coupled with his unstable housing situation. He had been accessing housing support in an attempt to stabilize his life, however, his wife’s passing quickly made him ineligible to continue residing in the couples shelter where they had been living. As a result, he was displaced without a viable housing option. This situation highlights the shortcomings in the very systems set up as safety nets for people who are homeless, which may exacerbate a person’s risk for overdose when coordination with mental health and substance use services are lacking.

#### Samantha

When “Samantha” met the team, she was unemployed, in an abusive relationship, and had inadequate access to early childhood development services for her autistic son. Samantha coped with these stressors by using substances. To address her drug use, the team first needed to help to address her existing stressors. As the team was helping Samantha find job fairs to attend, she disclosed that she was illiterate, something she felt deeply ashamed of; an important detail that she had never felt she could disclose to the City agency responsible for public assistance and job placement. The team refocused her job search towards positions that would not require reading and writing, setting Samantha up for greater success. They also explored the benefits that Samantha qualified for due to her son’s learning disability. Samantha was not interested in addressing her relationship with her abusive partner, but the team was able to bring stability to her life in concrete ways, increasing her sense of self-efficacy and her confidence as a parent. The PORT’s nonjudgmental approach laid the foundation of trust that made Samantha feel comfortable sharing her inability to read or write. This was her first experience with a provider in which she did not feel stigmatized.

#### Ezra

Lastly, “Ezra’s” case illustrates a PORT’s ability to sensitively repair relationships with healthcare providers. Ezra had been involved in several drug treatment programs, leaving one after being called a derogatory term by a staff member and leaving another because the programming requirements threatened his employment. Ezra had been repeatedly let down by the providers responsible for helping him, due to their stigmatizing behaviors and inability to see his need to maintain employment as critical to his stability. If he continued to miss work due to mandatory treatment program requirements he would lose income, which would have ripple effects and result in countless stressors, all contributing to the risk of overdose. The team worked with Ezra to help provide him access to the care he so desperately and actively sought, with providers who would treat him with respect, and help him without interfering with his employment.

## Limitations

One substantial limitation to note is the pilot phase of this program coincided with the early months of the COVID-19 pandemic. This may have significantly impacted clients’ willingness to receive a home visit or engage in services.

Another limitation to engagement is that PORT referrals are unaware that someone will be attempting to reach them, which makes initial engagement challenging. To address the lack of awareness within the communities we serve, the team has created palm cards to distribute while in the field. The team has also been asking for successfully reached referrals to notify their social networks about the program. To combat the lack of awareness of the NYSDOH PORT amongst other drug user health programs, the team has increased efforts to seek collaborations and partnerships with community-based organizations, public safety, and other public health partners. The management team has also prioritized presenting and attending relevant conferences and summits to raise key harm reduction stakeholders’ awareness of the PORT program.

PORT program engagement with law enforcement may be of concern to some, and our team would be remiss if we did not emphasize the complex history and relationships between law enforcement agencies such as the NYPD and PWUD. It is important to note that law enforcement’s role in the NYSDOH PORT program is only to provide referral information in a unilateral fashion; all outreach efforts carried out by the program are independent of NYPD operations.

## Conclusion

One particularly impactful element of the PORT may be attributed to the power of human connection itself. Receiving help from supportive individuals has been identified as “the most important factor to one’s personal recovery process” [12]. However, individuals with a SUD often have fewer social support network resources than those without SUDs [18]. Additionally, research conducted by Johansen et al. [12] Stott and Priest [20] suggest that social support can be an important factor in maintaining sobriety when combined with a focus on practical support, an essential component of post-overdose outreach work.

When housing and healthcare services exist in vacuums, individuals with complex needs suffer. Programs like PORTs are positioned to consider a person's overall needs and bridge gaps between systems, considering specific and immediate needs such as safe shelter, food, and healthcare. This becomes increasingly important when considering that the majority of unsheltered homeless in NYC are individuals living with mental illness or other severe health problems. However, this model is one that can arguably work for all individuals who use drugs by supporting safe drug use behaviors, not only those who may have more complex needs.

By cultivating human connection, a chief priority of the NYSDOH PORT, unique opportunities for intervention are presented. Even when an individual declines engagement in services, there remains a window to engage meaningfully, and the lived substance use experience of team members may serve to magnify such an opportunity. Although the use of peer support services to address specific social determinants of health associated with drugs is an area of emerging research, the positive effect of peer programs has been demonstrated in related work, such as HIV and other chronic illness care. Intervention models that utilize peer support have been associated with improvements in a range of substance use and recovery outcomes and have been found to improve the linkage of individuals to outpatient-based MOUD [3, 13].

It must be noted that a significant proportion of the individuals referred to the NYSDOH PORT were not able to be found. Over time, the team has come to understand that the willingness to be found may in itself be a social determinant of health, in part influenced by prior experiences by organizations serving this population. Risk factors for overdose are amplified by this inability to be located—whether by choice, in an effort to avoid contact with entities such as law enforcement or child protective services due to fears of adverse outcomes, or simply as a result of the often-transient nature of individuals who may lack stable housing, employment, and dependable social support. Due to prior negative encounters with such systems and associated stigma, many people may simply not want to be found. These risk factors, taken into consideration alongside social determinants of health, may provide a deeper understanding of PWUD, allowing for more informed decision-making when tailoring interventions to best meet their needs.

A comprehensive and flexible approach to working with PWUD, defined by person-led definitions of accomplishment, will only increase the likelihood of PORT success. As the needs of PWUD following a non-fatal overdose are varied, providing services beyond overdose response that address imminent needs and quality-of-life measures may reduce the likelihood of a subsequent fatal overdose, as supported by van Draanen et al. [22]. Researchers Olfson et al. [16], in a longitudinal study examining health outcomes in the year following a non-fatal overdose, found that individuals who had overdosed in the previous year died at approximately 24 times the rate of the general population. The causes of death were attributed to a wide range of substance use-associated mental health and medical conditions, highlighting the urgent need to coordinate medical, substance use, and mental health care immediately post-overdose [16]. PORTs can assess the myriad needs of the individual and tailor services accordingly rather than addressing one social determinant of health over another, ultimately resulting in individualized solutions to gaps in systems.

## Data Availability

The raw deidentified programmatic data used are available upon reasonable request.

## Abbreviations

PWUD: People who use drugs
SDOH: Social determinants of health
SEM: Socioeconomic marginalization
PORT: Post overdose response team
MOUD: Medication for opioid use disorder
NYSDOH: New York State Department of Health
NYPD: City of New York Police Department
HIPAA: Health Insurance Portability and Accountability
